# Defensins: Exploring Their Opposing Roles in Colorectal Cancer Progression

**DOI:** 10.3390/cancers16152622

**Published:** 2024-07-23

**Authors:** Hussein Sabit, Timothy M. Pawlik, Shaimaa Abdel-Ghany, Borros Arneth

**Affiliations:** 1 Department of Medical Biotechnology, College of Biotechnology, Misr University for Science and Technology, Giza P.O. Box 77, Egypt; hussein.sabit@must.edu.eg; 2 Department of Surgery, The Ohio State University, Wexner Medical Center, 250 Cunz Hall, 1841 Neil Ave. Columbus, OH 43210, USA; tim.pawlik@osumc.edu; 3 Department of Environmental Biotechnology, College of Biotechnology, Misr University for Science and Technology, Giza P.O. Box 77, Egypt; shaimaa.abdel-ghany@must.edu.eg; 4 Institute of Laboratory Medicine and Pathobiochemistry, Molecular Diagnostics, Hospital of the Universities of Giessen and Marburg (UKGM), Justus Liebig University Giessen, Feulgenstr. 12, 35392 Giessen, Germany; 5 Institute of Laboratory Medicine and Pathobiochemistry, Molecular Diagnostics, Hospital of the Universities of Giessen and Marburg (UKGM), Philipps University Marburg, Baldinger Str., 35043 Marburg, Germany

**Keywords:** colon cancer, colorectal cancer, CRC, defensins, mTOR, gut microbiota

## Abstract

**Simple Summary:**

This review explores the potential of human defensins, particularly HBD-1, in combating colorectal cancer (CRC) initiation and progression. Highlighting the need for early detection and novel CRC therapies, it examines HBD-1’s ability to inhibit the mTOR pathway, a key regulator of cell growth, positioning defensin-based treatments as a promising approach with evidence for suppressing cancer cell proliferation and tumor growth.

**Abstract:**

Colorectal cancer (CRC) represents a significant global healthcare burden, with a particularly concerning rising incidence among younger adults. This trend may highlight potential links between diet, gut microbiome, and CRC risk. Novel therapeutic options have been increasingly based on the understanding of molecular mechanisms and pathways. The PI3K/AKT/mTOR pathway, a crucial cell growth regulator, offers a promising target for CRC therapy. mTOR, a key component within this pathway, controls cell growth, survival, and metabolism. Understanding the specific roles of defensins, particularly human β-Defensin 1 (HBD-1), in CRC is crucial. HBD-1 exhibits potent antimicrobial activity and may influence CRC development. Deciphering defensin expression patterns in CRC holds the promise of improved understanding of tumorigenesis, which may pave the way for improved diagnostics and therapies. This article reviews recent advances in understanding regarding how HBD-1 influences CRC initiation and progression, highlighting the molecular mechanisms by which it impacts CRC. Further, we describe the interaction between defensins and mTOR pathway in CRC.

## 1. Introduction 

Colorectal cancer (CRC) ranks as the third most common cancer worldwide, impacting a significant portion of the global population worldwide as a major cause of cancer mortality [1]. CRC is on the rise among younger individuals (under 50), and previous reports have linked this trend to specific dietary habits and imbalances in the gut microbiome [2]. By analyzing how CRC varies geographically and over time, we can gain valuable insights into the varying risk factors, as well as better understand cancer control strategies. Advanced colon cancer presents a significant clinical challenge due to the high prevalence of metastatic disease and the emergence of chemotherapeutic drug resistance, resulting in dismal patient prognoses [3]. Colon cancer is a topic of great interest in the ongoing fight against cancer, and research has focused on developing more effective drugs to treat this disease [4].

The phosphatidylinositol 3-kinase (PI3K)/protein kinase B (AKT)/mammalian target of rapamycin (mTOR) pathway is a crucial signaling cascade governing cell growth, proliferation, and survival [5,6]. mTOR is part of two complexes (mTORC1 and mTORC2) that influence cell proliferation and metabolism. mTORC1 regulates growth through translation, transcription, and nutrient transport, while mTORC2 is independent of rapamycin and controls cell growth via cytoskeleton regulation [7]. mTOR is a serine/threonine kinase that can be affected by signals from nutrients, growth factors, and even the environment around the cell. These signals direct mTOR in managing various essential functions such as protein synthesis, cell growth, metabolism, regeneration, and aging [8].

Human defensins, categorized into α and β subfamilies, are produced by immune cells and epithelia, and play a vital role in immunity. α-defensins (e.g., Human Neutrophil Peptides 1-3 (HNP1-3), Human defensin 5-6 (HD5-6)) directly attack microbes, while β-defensins, expressed in mucosal tissues, combine anti-microbial and immune-modulating functions. This interaction exemplifies the innate immune system’s intricate and adaptable defense mechanisms [9,10,11]. Human β-defensin 1 (HBD-1) is a broadly effective antimicrobial peptide (AMP) that combats various pathogens, including Gram-negative *Escherichia coli* (*E. coli*). 

Deciphering the specific gene expression patterns of defensins in CRC holds promise for unlocking not only potential involvement in tumor development, but also more effective diagnostic tools and therapeutic strategies [12]. A greater knowledge of these patterns is particularly important considering the current lack of understanding regarding the roles of certain defensins, like defensin α5, in tumor formation and progression [13].

This editorial article examines recent breakthroughs in the understanding of defensins’ role in the initiation and progression of CRC, with a focus on the molecular mechanisms by which HBD-1 impacts the disease.

## 2. Colorectal Cancer 

Colorectal cancer (CRC) is a significant health concern in the United States, with over 153,000 new cases diagnosed in 2023. Additionally, CRC mortality has increased among specific demographic populations, highlighting the need for improved screening and targeted interventions for high-risk populations [14,15]. Screening remains paramount, as detection of early-stage disease improves patient outcomes. In addition to screening, the development of novel therapeutic strategies is equally important, as the combination of early diagnosis and innovative treatment options provides the best chance to improve CRC outcomes [16].

Colon cancer treatment typically involves a multimodal approach, which can involve surgical resection, as well as adjuvant/neoadjuvant therapy, targeted therapy, and immunotherapy. Other therapeutic approaches may include natural antibiotics such as defensin. In lab models, mice with induced colon cancer had reduced tumor size, increased apoptosis in cancer cells, and restored normal colon tissue architecture with HD-5; these data suggested that defensins may be a promising therapeutic strategy to treat CRC without harming healthy cells [17]. Targeting of HD-5 may extend also to treatment of ulcerative colitis from Crohn’s disease [18]. In fact, functional assays using CRC cell lines and xenograft mouse models demonstrated that HD-5 overexpression suppressed cancer cell proliferation, colony formation, and tumor growth. Mechanistically, HD-5 directly interacted with components of the PI3K signaling pathway, a well-known driver of cancer cell growth and metastasis. These findings suggest that HD-5 functions as a tumor suppressor in CRC and may represent a potential target for future therapeutic development [19].

## 3. LncRNA in CRC

Beyond a regulatory role in gene expression, lncRNAs are emerging as key players in CRC that may be important for therapeutic interventions [20]. Evidence suggests that lncRNAs are involved in various malignancies including colon [3,21] and breast cancer (BC) [22]. In this context, lncRNAs influence diverse cellular processes that are crucial for tumorigenesis such as cell proliferation, apoptosis, cell cycle, migration, invasion, and drug resistance. Interestingly, lncRNAs have been associated with improved CRC prognosis. Data from patients and in vivo immune cell analysis have demonstrated consistent expression of lncRNAs across diverse cell types. In turn, a risk score model using three specific lncRNAs (CYB561D2, LINC00638, and DANCR) has been proposed to predict patient outcomes [23]. The risk score correlated with the tumor immune microenvironment, suggesting a potential link between lncRNAs and the immune response. These data emphasize how immune-related lncRNAs may help to stratify patients relative to CRC prognosis, as well as possibly be targetable for therapeutic interventions [23]. Notably, B3GALT5-AS1, which acts as a tumor suppressor, is a downregulated lncRNA in CRC patients with a poor prognosis and liver metastasis [20]. Conversely, overexpression of B3GALT5-AS1 can inhibit cell proliferation while promoting migration and invasion, highlighting its complex and multifaceted role in cancer progression. These findings underscore the potential of lncRNAs as both diagnostic and therapeutic targets in CRC. The lncRNA KCNQ1OT1 plays a role in promoting CRC by influencing both cell growth and cell cycle progression. Upon silencing the lncRNA KCNQ1OT1, CRC cell growth and division were slowed [24]. Further analysis revealed changes in genes involved in DNA replication and cell cycle, providing evidence that KCNQ1OT1 is involved in tumor progression. Additionally, silencing KCNQ1OT1 suppressed tumor growth in a CRC mouse model. Collectively, these findings highlight KCNQ1OT1 as a potential novel CRC therapeutic target [24].

A recent study reported on a potentially novel tumor suppressor related to colorectal cancer (CRC): the long non-coding RNA (lncRNA) miR663AHG [25]. Interestingly, miR663AHG expression was much lower in CRC tumors compared with healthy tissues. Interestingly, downregulation of miR663AHG correlated with lower levels of another tumor suppressor, miR663a. Furthermore, patients with lower miR663AHG expression had worse clinical outcomes. In addition, in vivo mouse models demonstrated that miR663AHG had potent anti-cancer properties. Specifically, miR663AHG effectively inhibited the growth and spread of colon cancer cells. Mechanistically, the study suggested an interesting feedback loop: miR663AHG may bind directly to miR663a, preventing its degradation and ultimately boosting its tumor-suppressive effects. These findings strongly suggest that miR663AHG acts as a tumor suppressor in CRC, potentially through its interaction with miR663a [25]. 

ZNF667-AS1, a lncRNA, has also been demonstrated to be a tumor suppressor in CRC. Lower levels of ZNF667-AS1 have been noted in CRC tissues and cells compared with molecules promoting cancer (i.e., KIF5C). Increasing ZNF667-AS1 levels in CRC cells reduced their growth and spread while triggering cell death. Mechanistically, ZNF667-AS1 may interact with miR-523-3p, preventing it from inhibiting KIF5C. These data suggest that ZNF667-AS1 acts as a tumor suppressor by regulating this interaction, offering a potential new target for CRC therapies [26].

Cui et al. discovered a novel lncRNA, 495810, that was highly expressed in colon cancer and was associated with poorer patient prognosis. This lncRNA promotes cancer cell growth and inhibits cell death. Mechanistically, it may interact with a protein involved in energy production (PKM2), potentially fueling cancer cell growth. In turn, lncRNA 495810 may be a potential target for colon cancer therapy [27].

Collectively, the data strongly suggest that a diverse group of lncRNAs act as regulators in cancer progression. These lncRNAs potentially exert their influence by targeting various cellular signaling pathways, with the mTOR pathway emerging as a particularly interesting candidate for further investigation.

## 4. mTOR Pathway in CRC

**Figure 1:**
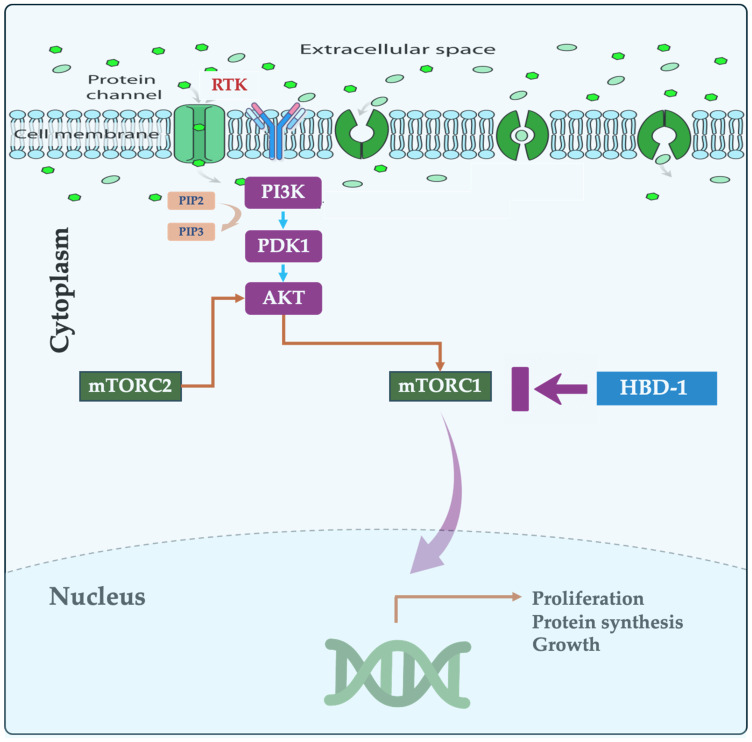
The Phosphoinositide 3-kinases/AKT/mammalian target of rapamycin (PI3K/AKT/mTOR) molecular pathway in colorectal cancer (CRC). This cell proliferation-related pathway can be blocked at different stages with different inhibitors. Human defensin beta-1 (HBD-1) can inhibit this pathway via downregulating mammalian targeting of rapamycin complex 1 (mTORC1).

The PI3K/AKT/mTOR signaling pathway is a highly conserved network that regulates critical cellular processes such as growth, proliferation, metabolism, and survival. Aberrant activation or dysregulation of this pathway has been implicated in the pathogenesis of numerous human diseases, including cancer, diabetes, and age-related disorders [28]. mTOR, a master regulator of cell functions like growth and metabolism, forms complexes that influence cell behavior. When the mTOR pathway is perturbed, the risk of various diseases, including CRC, increase [29]. In fact, uncontrolled activity of the mTORC2 pathway has emerged as a significant driver of human CRC progression acting as a cellular accelerator, promoting tumor growth and spread. Upon activation by growth factors, PI3K triggers phosphorylation and activation of AKT, which in turn activates mTOR. This pathway promotes cell growth and proliferation by stimulating protein synthesis, nutrient uptake, and cell cycle progression [30]. In contrast, cellular stressors or nutrient deprivation can inhibit this pathway, leading to cell cycle arrest and growth suppression. Dysregulation of the PI3K/AKT/mTOR signaling pathway is frequently observed in cancers, and leads to pleiotropic effects, including modulation of autophagy, epithelial–mesenchymal transition (EMT), apoptosis, chemoresistance, cell survival, and metastasis [31,32,33]. Thus, inhibiting this pathway has been shown to control the progression of various human cancers [30,34], highlighting its potential as a target for cancer therapy (Figure 1). A recent study by Park et al. (2023) identified a key genetic switch in the response of CRC patients to the drug SMI-4a. Patients with the unaltered (wild type) PIK3CA gene benefitted from treatment, while individuals with a mutated version exhibited resistance. These findings strongly suggest that PIK3CA may be a promising target for CRC treatment [35]. Understanding the role of mTORC2 in CRC, therefore, is important, as targeting this pathway has the potential to improve patient outcomes.

## 5. Human β-Defensins 

### 5.1. Structure and Function 

**Figure 2:**
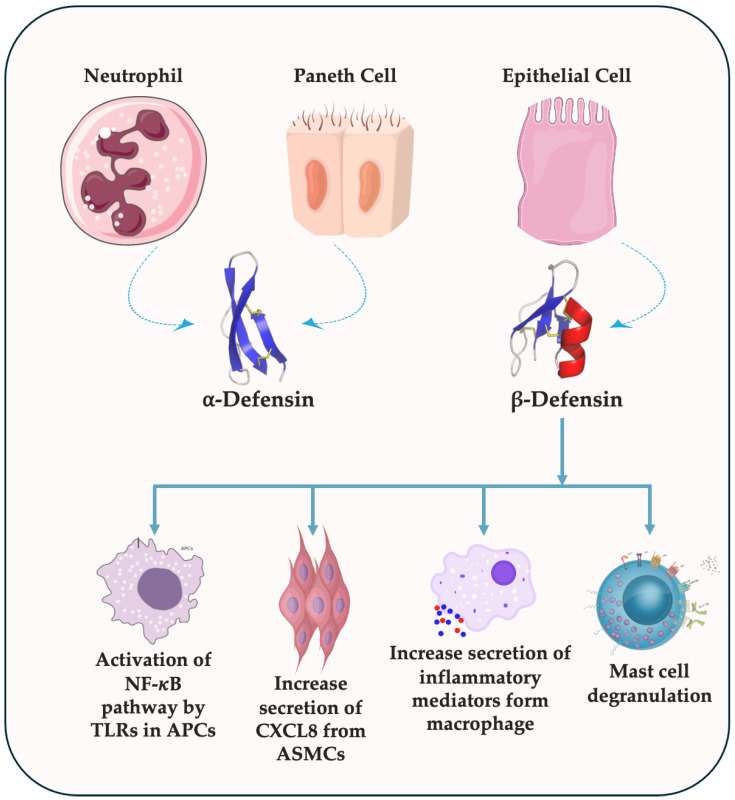
Secretion of α and β defensin. α-defensins are secreted from Paneth and Neutrophil cells, while β-defensins are secreted from epithelial cells.

Defensins, small proteins (18–45 amino acids) with a positive charge and special bonds that keep them stable [36], emerged on the scientific scene in 1985 with their groundbreaking discovery by Robert Lehrer. The initial defensins, which were isolated from rabbits, displayed impressive antibacterial and antiviral activity [37]. With a surge in defensin research, there were identifications of additional human defensin types over the subsequent decades. Specifically, there are three main defensin types (α, β, and θ) found in mammals; however, humans only have α and β. Defensins are made in a multi-step process starting as a pre-defensin with three parts: a signal segment, a pro-segment, and the mature peptide. The signal segment is quickly removed, leaving a pro-defensin. This pro-segment might help make defensins less toxic to our own cells. β-defensins have shorter pro-segments than α-defensins, possibly because they are produced differently. Defensins often link together as dimers or even larger groups, which may make them more effective at fighting bacteria and destroying membranes [38]. α-defensins are secreted by Paneth and neutrophil cells, while β-defensins are secreted by epithelial cells (Figure 2). 

Defensin genes are mostly grouped together on a specific chromosome, but some are scattered elsewhere in the genome [39]. α-defensins like HNP1-3 were the first to be characterized, followed by the discovery of HNP4, HD5, and HD6 [40]. A 2022 database of host defense peptides (HDPs) revealed a staggering number—over 3250. These natural antibiotics come from a wide range of organisms, with the vast majority (2521) being found in animals. Bacteria, archaea, protists, fungi, and plants contribute the rest. 

As a structurally diverse family of small, positively charged peptides, defensins play a crucial role in host defense. In particular, defensins exhibit potent antimicrobial activity against bacteria, viruses, and fungi [41]. These peptides are categorized into subfamilies based on the unique arrangements of three disulfide bonds formed by six cysteine residues within their structure. The first identified α-defensins were isolated from rabbit granulocytes [37]. Shortly thereafter, human α-defensins, initially named human neutrophil peptides (HNP 1–3), displayed broad-spectrum antimicrobial activity, leading to the coining of the term “defensin” for this family. Later research identified HNP-4, a neutrophil α-defensin with a distinct amino acid sequence compared with the first three HNPs. Interestingly, mice lack neutrophil α-defensins altogether [42]. Mice, however, possess α-defensins produced by Paneth cells in the intestine, known as cryptdins. While both human and murine α-defensins exhibit antimicrobial properties, cryptdins are markedly more abundant in mice versus human neutrophil α-defensins [43]. Overall, there is fascinating diversity of defensin subfamilies with a broad distribution across different mammalian species.

β-defensins exhibit a diverse gene family across vertebrates. Their genes typically have two exons, with the first encoding a precursor peptide and the second containing the signature six-cysteine motif [44]. Recent genome sequencing has revealed species-specific clusters of β-defensin genes, ranging from 14 in chickens to 48 in humans and mice [45,46]. Interestingly, specific amino acid positions are under evolutionary pressure, potentially leading to further functional diversification [47].

### 5.2. Defensins in Cancer

Traditionally known for fighting microbes [48,49] and exhibiting various immune functions [49], defensins are now being studied in cancer. Defensins have been identified in various cancers [13,50] and act as versatile immune modulators in the tumor microenvironment [51,52]. Defensins can attract immune cells like T cells and dendritic cells [49], suggesting a broader role beyond just fighting infection. Mechanistically, defensins inhibit cancer cells via specifically attaching to areas on the tumor cell membrane rich in phosphatidylserine (PS). This binding sets off a chain reaction within the cancer cell, ultimately leading to its self-destruction and potential tumor shrinkage [53]. This distinct mechanism offers promise for increased selectivity and the overcoming of resistance [54].

β-Defensin 1 (HBD-1), located on chromosome 8p in the human genome [55], appears to act as a tumor suppressor in various cancers [56,57,58]. The expression of HBD-1 is generally downregulated in tumor, as opposed to healthy, tissues across a variety of cancers including colorectal, liver, and skin; in turn, HBD-1 appears to function as a tumor suppressor [59,60]. The downregulation of HBD-1 correlates with markers of poor prognosis and tumor progression. Conversely, factors that increase HBD-1 expression such as inhibiting EGFR in CRC, seem to have an anti-tumor effect. These findings suggest HBD-1 may play a role in suppressing cancer cell growth, migration, and invasion through pathways impacting cell signaling and matrix remodeling [12,61]. Interestingly, some chemotherapeutic drugs suppress HBD expression in cancer cell lines; other data suggest that vincristine and doxorubicin can upregulate HBD-1 and HBD-3/4, respectively. These findings highlight the complex and sometimes opposing roles that HBDs can play in the context of cancer [62,63,64].

Other family members of the HBD family appear to play a pro-cancerous role, including β-Defensin-3 (HBD-3). HBD-3 over-expression has been linked to migration, invasion, and cell death in various cancers including Lewis lung carcinoma and CRC [65,66]; other studies have suggested a pro-cancerous effect [61,67]. HBD-3 may contribute to cancer development by activating signaling pathways crucial for cell proliferation, although the interplay between defensins and the tumor microenvironment is complex [61,67,68]. For example, HBD-2 may suppress tumors when downregulated, yet potentially promote tumor growth when upregulated [66]. This complexity underscores the need for further research to understand β-defensin regulation in different cancers.

Defensin α 5 (HAD-5) has also been demonstrated to act as a tumor suppressor in CRC. Overexpression of HAD-5 inhibited CRC cell proliferation, colony formation, and tumor growth in mice [19]. Mechanistically, HAD-5 binds to PI3K complex subunits, affecting downstream signaling and hindering cell growth and metastasis. Therefore, HAD-5 may serve as a therapeutic target for CRC [19]. Table 1 highlights the fate of different defensins in different types of cancer.

Defensins, both α and β, have altered expression in cancer, versus healthy, tissue. Large-scale analysis of cancer data suggests a link between interferon (IFN) activity, defensin genes, and resistance to immunotherapy. Many cancer types frequently lose IFN and defensin genes through deletions, and this loss has been correlated with worse patient outcomes [12]. One study identified a ratio (HDI/HDD) of deleted IFN genes to deleted defensin genes, with higher ratios associated with more aggressive cancers like brain and pancreatic tumors. Conversely, cancers with a higher proportion of deleted defensin genes (HDD/HDI) like prostate and CRC may respond better to immunotherapy [12]. Intermediate ratios were noted in other cancers like breast and lung. Beyond deletions, gene expression analysis suggests that deleted IFN and defensin genes lead to activation of cancer-promoting pathways and suppression of immune response pathways. These data imply that defensins have a tumor-suppressive effect that is lost with deletion. The specific immune effects vary between and within the α- and β-defensin families [76].

The role of α-defensins in cancer is complex. While α-defensin 5 (DEFA5) suppresses gastric cancer growth, α-defensin 5 and 6 (DEFA5/6) are elevated in colorectal cancer. DEFA5 might be linked to a better prognosis, while DEFA6 might be associated with a worse prognosis. These findings suggest α-defensins have diverse functions in different cancers, potentially serving as biomarkers or therapeutic targets [9,77]. 

### 5.3. β-Defensins as Proinflammatory Mediators

Defensin production adapts to infection, offering both pre-emptive defense and a response to a stimulus. Beyond fighting microbes, defensins are immune regulators and cell recruiters [78], although their origin is unclear [79]. Generally produced by immune cells like neutrophils and epithelial cells lining our body surfaces, defensins are natural defenders and play a role in innate immunity in the gastrointestinal tract. Researchers have been particularly interested in understanding how defensins function within the body’s natural defenses, as well as how their structure relates to their effectiveness at facilitating the killing of microbes, as well as their potential as future therapeutic drugs [80]. These peptides, expressed differently in various cancers, can directly kill tumor cells and even attract and activate immune system soldiers like T cells and antigen-presenting cells. The induced multi-pronged attack suggests defensins may be a valuable tool to boost the body’s natural defenses against cancer, potentially enhancing the effectiveness of immunotherapy [49]. 

Defensins act as messengers to trigger and strengthen the body’s overall immune response, including both cell-mediated (Th1) and antibody-based (Th2) arms. Giving defensins along with antigens to mice boosts their immune response by increasing the production of immune signaling molecules (cytokines) from both Th1 and Th2 cells [81]. Additionally, defensins work in conjunction with other immune cells. For instance, defensins released by skin cells can activate receptors on neutrophils, leading to the production of molecules that further enhance infection resistance [82,83]. Furthermore, defensins can prevent autoimmune diseases by suppressing the production of inflammatory factors and promoting regulatory T-cell responses [84,85].

β-defensins bind to receptors on immune cells like CCR6, CCR2, and TLRs, triggering a coordinated response. This response includes maturing antigen-presenting cells for a stronger adaptive immune response, as well as stimulating the production of proinflammatory molecules by immune cells. β-defensins also extend the lifespan of neutrophils, all working together to combat infections. These receptor-mediated activities solidify β-defensins as a critical link between innate and adaptive immunity [86,87]. Defensins actively recruit immune system allies like T cells and immature dendritic cells, and also stimulate immune cells to fight cancer by expressing pro-inflammatory signals [49]. Additionally, defensins can directly enter macrophages and inhibit the production of inflammatory molecules [88,89].

While the immunomodulatory role of defensins is becoming clearer, many questions remain. Researchers are still trying to understand how these peptides activate numerous immune pathways and whether defensins act as sensors, activators, or effectors in immune regulation. Further research on these mechanisms is crucial to develop defensins as potential therapeutic targets.

### 5.4. Defensins and the Gut Microbiota 

The trillions of microbes residing within our gut, primarily bacteria but also viruses and fungi, form a complex ecosystem known as the gut microbiota (GMB) [2,90]. This microbial community interacts closely with our bodies, impacting both metabolism and immune function, and even extending its influence on brain health. When disrupted or unbalanced (dysbiosis), the GMB can trigger inflammation, potentially leading to severe disorders [91].

Newborn babies acquire their initial GMB from their mothers during birth. Over time, exposure to environmental microbes further shapes this microbial community. The gut microbiota undergoes a transformation throughout life, with the early stages dominated by Bifidobacterium and Lactobacillus species [92]. In healthy adults, a balanced community of Firmicutes and Bacteroidetes takes hold [90]. However, factors like dietary changes and antibiotic use can disrupt this delicate balance, leading to a state known as dysbiosis.

GMB stimulates innate lymphoid cells (ILCs) which in turn induce pancreatic cells to produce β-defensin 14 (mBD14). mBD14 promotes the development of regulatory immune cells, ultimately preventing autoimmune destruction of insulin-producing pancreatic cells in mice. Interestingly, dysbiosis in the GMB and a specific genetic variant affecting an immune receptor were linked to the observed deficiencies in mBD14 production in diabetic mice [93]. This dysbiosis may be due to unhealthy diet intake, which leads to decreased fecal β-defensin-3 levels; thus, monitoring fecal β-defensins may be a useful tool for microbiota-directed therapies [94].

Meanwhile, radiation can reduce the expression of α-defensin peptides produced by Paneth cells in the intestine [95]. α-defensin peptides help maintain a healthy GMB composition. Radiation-induced decreases in α-defensin were associated with changes in the GMB, increased intestinal permeability (leaky gut), and the presence of bacterial toxins in the bloodstream. These findings suggest that Paneth cell dysfunction is a key factor in radiation-induced gut issues, and administration of HD5 has potential as a preventative and therapeutic approach [96,97].

HD5 levels were decreased markedly in elderly individuals compared with middle-aged adults. Furthermore, HD5 levels have been negatively correlated with specific bacterial groups that increase with age, as well as a positive correlation with bacterial groupings that decreased with age. These findings suggest that declining HD5 levels with age may be a contributing factor to the changes observed in gut bacteria of elderly individuals, potentially increasing their risk of disease [98]. In another study, researchers examined the impact of defensins on gut microbiota composition. While defensins appeared to play a role in shaping the gut bacteria of young mice, their influence seemed less pronounced in adult mice with a mature gut microbiome. The study identified a new role for defensins in adult mice: protection against diet-induced complications such as worsened glucose metabolism [99]. Overall, these studies suggest that defensins play a more nuanced role in gut health. Defensin influence on gut bacteria composition may be more significant during early life, but defensins continue to be important for protecting against diet-related metabolic issues into adulthood.

Research continues to shed light on the complex relationship between defensins and the gut microbiome. Understanding how defensins influence specific bacterial populations may lead to new therapeutic approaches for gut-related diseases. Future studies will need to explore how to manipulate defensin levels or harness their power to promote a healthy gut microbiome.

## 6. Conclusions

Beyond their well-established antimicrobial activity, defensins exhibit a surprising versatility in regulating cancer and gut health. Defensins directly eliminate cancer cells and recruit immune cells to tumors. While in the gut, defensins help to maintain a balanced microbiome. Functioning as immune messengers, defensins bridge innate and adaptive immunity by activating and prolonging the lifespan of immune cells. Notably, deficiencies in defensins have been linked to gut disorders and worse cancer prognoses, highlighting their potential as therapeutic targets. Future research will need to examine the manipulation of defensin levels, as well as the harnessing of their immunomodulatory properties, to promote health and combat disease. Unveiling the intricate molecular mechanisms by which defensins function could unlock a new frontier of therapeutic targets for various cancers, including CRC. By manipulating defensin activity or mimicking their properties, researchers envision novel strategies to combat these diseases.

## Figures and Tables

**Table 1:** The fate of different defensin in different types of cancer.

Cancer Type	HBD-1	HBD-2	HBD-3	Source	Cases/Control	Ref.
Cervical	--	Upregulated	Upregulated	Patients	37/22	[62]
Oral	Downregulated	Downregulated	Upregulated	Cell lines	16/15	[69]
Lung	Upregulated	Upregulated	--	Patients	56/46	[70]
Skin	Downregulated	Upregulated	--	Patients	22/27	[71]
Esophagus	--	Upregulated	--	Patients	58	[72]
Liver	Downregulated	--	--	Patients	733/656	[61]
Kidney	Downregulated	--	--	Patients	48	[73]
Colon	Downregulated	Downregulated	Downregulated	Patients	40	[74]
Prostate	--	--	--	Patients	100	[73]
Tonsil	--	Downregulated	--	Patients	8/8	[75]
